# Mental illness stigma and associated factors among Arabic-speaking refugee and migrant populations in Australia

**DOI:** 10.1186/s13033-023-00580-z

**Published:** 2023-05-03

**Authors:** Ritesh Chimoriya, Yaser Mohammad, Russell Thomson, Cheryl Webster, Rachel Dunne, Michaels Aibangbee, David Ip, Shameran Slewa-Younan

**Affiliations:** 1 School of Medicine, Western Sydney University, Campbelltown, Australia; 2 Translational Health Research Institute, Western Sydney University, Campbelltown, Australia; 3 School of Computer, Data and Mathematical Sciences, Western Sydney University, Penrith, Australia; 4 Anglicare, Anglican Community Services, Baulkham Hills, Australia; 5 Centre for Mental Health, Melbourne School of Population and Global Health, University of Melbourne, Melbourne, Australia

**Keywords:** Arabic-speaking, Refugees, Migrants, Mental health, Mental health literacy, Mental illness, Stigma

## Abstract

**Background:**

Arabic-speaking refugee and migrant populations form a significant proportion of Australia’s population. Despite high levels of psychological distress among Arabic-speaking populations, low uptake of mental health services has been demonstrated. Evidence suggests poor levels of mental health literacy (MHL) and high levels of stigmatising attitudes among Arabic-speaking populations, which may act as barriers to help-seeking behaviours. This study aimed to explore the relationships between measures of mental illness stigma, socio-demographic factors and psychological distress, as well as to determine the factors associated with MHL (i.e., correct recognition of mental illness and knowledge of causes) among Arabic-speaking refugee and migrant populations in Australia.

**Methods:**

Participants were recruited from non-government organisations in Greater Western Sydney that provided support services to Arabic-speaking migrants and/or refugees. As this study is nested within an interventional pilot study evaluating a culturally tailored MHL program, only the pre-intervention survey responses for 53 participants were utilised. The survey measured key aspects of MHL (i.e., recognition of mental illness, knowledge of causes), levels of psychological distress (using K10 scale), and stigmatising attitudes towards mental illness (using Personal Stigma Subscales and Social Distance Scale).

**Results:**

The Personal Stigma subscale of ‘Dangerous/unpredictable’ was strongly positively correlated with participants’ K10 psychological distress scores and strongly negatively correlated with years of education completed. There were moderate negative correlations between two Personal Stigma subscales (‘Dangerous/unpredictable’ and ‘I-would-not-tell-anyone’) and the length of stay in Australia. Being female was associated with an increase in personal stigma demonstrated by higher scores for ‘I-would-not-tell-anyone’ subscale than males. Similarly, increase in age was associated with a decrease on scores of the personal stigma ‘Dangerous/unpredictable’.

**Conclusions:**

While future research with larger sample size are needed, the study findings can be considered as adding to the evidence base on mental illness related stigma in Arabic-speaking populations. Further, this study provides a starting point in developing the rationale for why population sub-group specific interventions are required to address mental illness stigma and improve MHL among Arabic-speaking refugee and migrant populations in Australia.

## Background

Australia is one of the most culturally and linguistically diverse countries in the world, with over 7.6 million migrants residing in Australia in 2020 and 29.8% of its population born overseas [1]. Most migrants in Australia arrive through the Family Stream (i.e., migration of family members of Australian citizens or permanent residents) and through Australia’s Refugee and Humanitarian Program [2]. Arabic-speaking population form a large proportion of Australia’s refugee population, with the majority of offshore humanitarian visas in the period of 2015 to 2019 granted to those born Iraq and Syria due to the ongoing conflicts [2, 3]. According to the Australian Bureau of Statistics (ABS) 2016 census, Arabic-speaking refugee and migrant populations represent 1.4% of the total Australian population, and Arabic is the third most spoken language in Australia following English and Mandarin [4].

Refugees and migrant populations can face several stressors associated with migration, including separation from family and support networks, social isolation, racism, language barriers, and financial hardship, all of which can contribute to poor mental health outcomes [5, 6]. Particularly for refugee populations, pre-migration experiences characterised by exposure to traumatic events, combined with post-migration stressors, can put them at a high risk of developing mental health problems [7–9]. There is substantial evidence indicating that refugee populations demonstrate high levels of psychological distress and are at a higher risk of developing mental disorders, especially post-traumatic stress disorder (PTSD) and major depressive disorder [10, 11]. Iraqi refugees resettled in Western countries have been shown to have significantly higher prevalence of PTSD (8–37.2%) and depression (28.3–75%), compared to the general Australian population as well as the general Iraqi population [6, 12]. A recent systematic review on Syrian refugees resettled in high-income Western countries also found elevated rates of PTSD, depression and anxiety, with the total pooled prevalence rates of 31%, 31% and 40%, respectively [13].

Despite evidence on the high levels of psychological distress among Arabic-speaking populations, especially among those with a refugee or asylum-seeker background, low uptake of mental health services for psychological issues has been demonstrated [14–16]. While evidence suggests low levels of professional help-seeking behaviours and attitudes among Arabic-speaking refugee and migrant populations [15, 17, 18], this could be also attributed to their preference for seeking help for mental health difficulties from informal sources such as family, friends, and religious leaders [15, 19, 20]. Nonetheless, an essential concept that may be related to help-seeking behaviours is mental health literacy (MHL), defined as “knowledge and beliefs about mental disorders which aid their recognition, management or prevention” [21]. MHL encompasses “the ability to recognise specific disorders; knowing how to seek mental health information; knowledge of risk factors and causes, of self-treatments and of professional help available; and attitudes that promote recognition and appropriate help-seeking” [21].

The ability to recognise mental health problems or specific disorders forms a major component of MHL [21]. Poor levels of MHL have been reported for Arabic-speaking communities in Australia, with low levels of recognition of mental illness such as PTSD and complex beliefs and knowledge about mental illness, which may act as barriers to help-seeking [22, 23]. Arabic-speaking individuals also have been observed to perceive depression and anxiety as common experiences, often attributed to social factors such as unemployment and financial hardship, and they do not label them as mental illnesses or seek treatment [23]. Furthermore, lower levels of MHL have been linked with stigma [24], which refers to an interaction of negative cognitions, emotional reactions, and behaviours [25]. Negative attitudes toward mental illness can impede recognition of mental illness as well as help-seeking for mental health issues [25, 26]. Stigmatising attitudes towards mental illness has been noted to be prominent in Arabic-speaking refugee and migrant populations [23, 27]. “Mental health problems” and “mental illness” are greatly stigmatised among Arabic-speaking populations, and these terms usually provoke a desire to distance oneself from an individual with a mental health problem [28]. Among Arabic-speaking populations, high levels of reluctance to engage with individuals with mental illness, across varied social situations and relationships such as family, friends, and neighbours, have also been observed [29, 30]. Many Arabic-speaking individuals have been found to experience and express their psychological distress as somatic symptoms, such as digestion issues and headaches, and they consider the symptoms to be linked with physical illness rather than mental illness [31]. Expression of such distress as physical illness has been identified to provoke fewer stigmatising responses from other community members [32].

Beliefs about the causes of mental health problems and knowledge of risk factors and causes of mental illness are also central to MHL [21]. While some Arabic-speaking individuals endorse notions consistent with biomedical models of mental illness (e.g., chemical imbalance), stigmatising beliefs regarding the origins and meaning of mental illness are prevalent among Arabic-speaking populations, including among health professionals [33–35]. Commonly reported stigmatising beliefs include mental illness as originating from personal weakness [36], individuals with mental illness being dangerous and unpredictable [29, 37], and mental illness as originating from supernatural forces [29]. Supernatural and religious attributions of mental illness, such as God’s punishment, black magic, and satanic powers, are also widespread among Arabic-speaking populations living in Arab countries as well as Australia [23, 29, 34, 38]. Socio-demographic factors have also been found to play a role in the variation of beliefs about mental illness, with more females endorsing “those who are not very religious” as a risk factor for developing PTSD, as compared to males [33]. Stigmatising beliefs and attitudes can be a significant barrier to help-seeking, particularly when seeking counselling is not accepted within the Arabic-speaking community and to seek such assistance can lead to the individual being marked as “mad”, as was reported in an Australian qualitative study [23].

Findings from prior studies indicate poor levels of MHL [22, 23] and high levels of stigmatising attitudes among Arabic-speaking populations [23, 27], which may act as barriers to professional help-seeking intentions and behaviours. While there are prior studies that have explored MHL amongst Arabic-speaking community in Australia [8, 22, 39], research exploring factors associated with mental illness stigma is scarce. A recent study among Arabic-speaking religious and community leaders in Australia emphasised the need for future research to further elucidate stigma among Arabic-speaking populations [20]. Gaining a comprehensive understanding of mental illness stigma among Arabic-speaking refugee and migrant populations is pivotal to addressing their mental health needs and to facilitate the uptake of mental health services. An understanding of whether socio-demographic factors and levels of psychological distress play a role in the stigmatising attitudes and beliefs towards mental illness held by Arabic-speaking populations is essential for the development of early interventions. As such the current study aimed to explore the relationships between measures of mental illness stigma and various associated factors such as socio-demographic factors and measure of psychological distress, as well as to determine the factors associated with MHL in terms of correct recognition of mental illness and knowledge of its causes among Arabic-speaking refugee and migrant populations in Australia.

## Methods

### Study design and participants

The current study is nested within an interventional pilot study, which aimed to evaluate a culturally tailored MHL Program designed to enhance MHL of the Arabic-speaking refugee populations residing in South Western Sydney, Australia [8]. Participants were recruited from non-government organisations located in Greater Western Sydney that provided support services to Arabic-speaking migrants and/or refugees. Eligible participants were Arabic-speaking women and men aged 18 years or above, who had arrived in Australia as a migrant, and/or under the Humanitarian Migration Program.

The participants attended and participated in the MHL Program, which comprised of three-hour weekly sessions held over a four-week period, delivered in Arabic by experienced bilingual health educators and/or mental health clinicians. The intervention has been described in detail in a previous publication [8]. The participant information sheet, consent forms, and the survey forms were provided to the participants in Arabic. The participants were asked to complete surveys at three time points, pre-intervention, post-intervention, and three months follow-up. In the current study, only the survey responses for the 53 participants who completed the pre-intervention survey and participated in the intervention were utilised. Ethics approval was attained from Western Sydney University Human Research Ethics Committee (approval number H12707). All participants provided written consent to participate in the study prior to completing the pre-intervention survey.

### Measures

The self-report survey assessing the key aspects of MHL utilised in the present study is based on the survey developed by Jorm et al. [21], which was further adapted for refugee populations in a prior study by Slewa-Younan et al. [22]. In addition, the Kessler Psychological Distress Scale (K10) was used to assess psychological distress [40]. The socio-demographic characteristics of the study participants were also collected.

### Recognition of mental health problem as PTSD

The use of vignettes to measure MHL has been previously validated [41], and the vignette utilised in the current study has also been used in several prior studies [10, 17, 22, 42–44]. A culturally valid vignette depicting a fictional Iraqi refugee, either named ‘Dawood’ or ‘Miriam’ based on the gender of the participant, was provided to the participants. It was ensured that Dawood/Miriam’s character met the criteria for PTSD as outlined in the 5th edition of the Diagnostic and Statistical Manual of Mental Disorders (DSM-5) [45]. The vignette was used to assess the participants’ ability to recognise Dawood/Miriam’s mental health problem as PTSD. Participants were asked ‘What would you say is Dawood/Miriam’s main problem?’, and they were presented with six possible options with the requirement to select only one option. Only the response ‘Fear/PTSD/Stress Related Disorder’ was coded as ‘correct’, while other responses (e.g. weak character, physical condition) were coded as ‘incorrect’.

### Stigmatising attitudes towards mental illness

Stigmatising attitudes towards mental illness were assessed utilising the modified Personal Stigma in Response to Mental Illness Scale [44, 46, 47]. The personal stigma scale assessed the participants’ personal attitudes towards the character described in the vignette (i.e., Dawood/Miriam). Participants were asked to respond to seven statements assessing personal stigma using a five-point Likert scale, ranging from 1 (‘strongly disagree’) to 5 (‘strongly agree’). The statements were divided into three components: ‘I-would-not-tell-anyone’, ‘Weak-not-sick’, and ‘Dangerous/unpredictable’ subscales, as utilised and validated in prior studies [41, 48–50]. The ‘I-would-not-tell-anyone’ subscale comprised one item that focused on the belief that it is better not to tell others if they had a similar problem (e.g., “You would not tell anyone if you had a problem like Dawood/ Miriam’s”). The ‘Weak-not-sick’ subscale comprised items that focused on the belief that the person is not legitimately ill, they can control their problem, and their problem is a sign of weakness (e.g., “Dawood/Miriam’s problem is not a real medical illness”). The ‘Dangerous/unpredictable’ subscale comprised items that focused on the belief that someone with mental problems is dangerous or unpredictable (e.g., “Dawood/Miriam’s problem make him/her unpredictable”). Higher scores indicate higher levels of personal stigma for each subscale.

Stigmatising attitudes towards mental illness were also assessed using five statements from the Social Distance Scale developed by Link et al. [51], and as used in prior studies [20, 44]. The Social Distance Scale assessed the willingness of the participants to spend time with Dawood/Miriam through several hypothetical relationships (e.g., friend, neighbour, colleague) [51, 52]. Participants were asked whether or to what extent they would be pleased “to move next door to Dawood/Miriam” or “to have Dawood/Miriam marry into your family” among others. The participants were asked to respond to the five statements assessing social distance using a four-point Likert scale ranging from 1 (‘Yes, definitely’) to 4 (‘Definitely not’). The total social distance score was calculated as the sum of the responses to each item, with higher scores indicating greater desire for social distance.

### Causal beliefs about developing mental illness

The participants’ beliefs about the causes for developing mental illness was assessed using a question about the possible causes of Dawood/Miriam’s problem. The participants were asked: “How likely do you think each of the following is a factor in this sort of problem developing in anybody?”. Participants were presented with 11 possible causes that could have led to Dawood/Miriam’s problem. However, for purposes of this study, only causal beliefs of the illness being a “Punishment from God” or due to “Being a person with a weak character” were considered due to most closely aligning to religious beliefs and/or stigmatising views and have previously been found to be commonly selected in other studies of MHL of Arabic-speaking samples [33, 42]. The participants were asked to rate each item as ‘very likely’, ‘likely’, or ‘not likely’, which for analysis were then collapsed into 1 (‘likely’, by combining responses for ‘likely’ and ‘very likely’) and 0 (‘not likely’).

### General psychological distress

The participant’s levels of general psychological distress at the pre-intervention stage were measured using K10, which is a scale of non-specific psychological distress developed by Kessler et al. [40]. The 10-item questionnaire contained questions related to anxiety, negative mood, and emotional states in order to quantify the frequency and severity of the symptoms during the past four weeks [53]. The items were scored on a five-point Likert scale ranging from 1 (‘none of the time’) to 5 (‘all of the time’). Individual scores of the items were summed to attain a total score ranging from 10 to 50, with higher scores indicating higher levels of psychological distress. Based on the total K10 score, the levels of psychological distress were categorised as: low-mild (10–21), moderate (22–29), and severe (30–50) [40]. Attributed to its strong psychometric properties, K10 is frequently used in health surveys and across diverse study populations [53], including resettled refugee populations in Australia [10, 43].

### Statistical analysis

The Statistical Package for Social Sciences (SPSS 27.0 for Mac, IBM Corp., Armonk, NY, USA) was used for the statistical analysis [54]. Three types of statistical methods were used. Bivariate correlation was used to explore the relationships between stigma scale scores. Multiple linear regression models were used to test which of the demographic variables are best predictors of the stigma scale scores. Multiple logistic regression models were used to test which of the demographic variables and scales are best predictors of correct recognition of the PTSD.

A non-parametric Kendall’s Tau-b correlation was performed to determine the relationships between the stigma scales (i.e., Personal Stigma Subscales and Social Distance Scale), as well as the relationships between the stigma scales and socio-demographic variables and K10 psychological distress scale.

Standard multiple regression analyses were performed to test whether socio-demographic variables predicted the stigma scale scores, with age and gender as independent variables and stigma subscales as the dependent variable. For the multiple linear regression models, percentage variance was presented based on R^2^. To account for departures from normality, the standard errors of the beta coefficients, *p*-values and confidence intervals were calculated using bootstrapping based on 1000 samples.

Binary logistic regression analyses were performed to measure the effect of socio-demographic factors, psychological distress, and stigmatising attitudes on the ability to correctly recognise Dawood/Miriam’s mental health problem as PTSD. Correct recognition of PTSD was entered as the dependent variable and socio-demographic factors, psychological distress scale and the stigma subscales were entered as independent variables.

A series of binary logistic regression analyses were also performed to determine whether socio-demographic factors, psychological distress, and stigmatising attitudes influenced the likelihood of selecting the two causal beliefs as ‘likely’ for developing a problem like Dawood/Miriam. For each logistic regression analysis, a stigma subscale (e.g., ‘I-would-not-tell-anyone’), a socio-demographic factor, or K10 psychological distress scale was entered as the independent variable and a cause (e.g., ‘Being a person with a weak character’) was entered as the dependent variable. The results of the logistic regression analyses were presented as odd ratios. Benjamini-Hochberg method with a false discovery rate of 25% was used for adjusting for multiple testing. *P*-values of < 0.05 were considered as statistically significant.

## Results

### Socio-demographic characteristics

Table 1 outlines the socio-demographic characteristics of the study participants. A total of 53 participants who completed the pre-intervention survey were included in the current study. The majority of the participants were females (66%) and were married (73.6%). The mean age of the participants was 52.3 years (SD 11.7), and they had completed an average of 9.8 years (SD 4.0) of education. A large proportion of participants were from Iraq (73.6%), followed by Syria (18.9%) and Lebanon (7.5%). The main spoken language at home was Arabic (88.7%), followed by Assyrian (7.5%) and Chaldean (3.8%). The participants’ average length of stay in Australia was 8.1 years (SD 9.1), and the participants had arrived in Australia as an asylum seeker (41.5%), on a refugee visa (32.1%), or an immigrant (22.6%). The mean K10 Psychological Distress score was 30.7 (SD 11.7); 30.2% of participants had a K10 score in the low-mild range (10–21), 9.4% in the moderate range (22–29), and 58.5% in the severe range (30–50).

**Table 1:** Socio-demographic characteristics of the study participants

Characteristics	Pre-Intervention (*n* = 53) *	
	*N*	Valid %
Gender		
Male	18	34
Female	35	66
Age (years), mean (SD)	52.3 (11.7)	
Country of Origin		
Syria	10	18.9
Iraq	34	73.6
Jordan	2	3.8
Lebanon	4	7.5
Egypt	2	3.8
Kuwait	1	1.9
Marital Status		
Never married	3	5.7
Married	39	73.6
Fiancé/Partner	1	1.9
Divorced	4	7.5
Widowed	6	11.3
Language spoken at home		
Arabic	47	88.7
Assyrian	4	7.5
Chaldean	2	3.8
Length of stay in Australia (years), mean (SD)	8.1 (9.1)	
Arrival status in Australia		
Refugee	17	32.1
Asylum seeker	22	41.5
Immigrants	12	22.6
Years of education completed (years), mean (SD)	9.8 (4.0)	
K10 Psychological Distress		
Low-mild (10–21)	6	30.2
Moderate (22–29)	5	9.4
Severe (30–50)	31	58.5

* Due to missing or incomplete data for some items, the total of the categories might not always add up to the total number of participants

### Correlations between the stigma scales

Kendall’s Tau-b correlations of Personal Stigma Subscales and Social Distance Scale are presented in Table 2. There was a moderate, positive correlation between ‘I-would-not-tell-anyone’ and ‘Dangerous/unpredictable’ subscales, which was statistically significant (*τ*_b_ = 0.25, *p* = 0.014). Means and standard deviations for the stigma scale scores were: I-would-not-tell-anyone (M = 3.2; SD = 1.3); Weak-not-sick (M = 12.4; SD = 2.5); Dangerous/unpredictable (M = 10.2; SD = 3.5); Social Distance (M = 11.5; SD = 3.6).

**Table 2:** Kendall’s Tau-b correlations of Personal Stigma Subscales and Social Distance Scale

Variable	I-would-not-tell-anyone	Weak-not-sick	Dangerous/unpredictable
I-would-not-tell-anyone			
Weak-not-sick	0.03		
Dangerous/unpredictable	0.25*	0.11	
Social distance	0.06	-0.11	0.04

* Correlation is significant at *p* < 0.05 (2-tailed)

### Correlations between stigma scales, socio-demographic variables and K10 scale

Kendall’s Tau-b correlations of the stigma scales, socio-demographic variables, and K10 psychological distress scale showed strong, positive significant correlation between ‘Dangerous/unpredictable’ subscale and K10 psychological distress scale (*τ*_b_ = 0.32, *p* = 0.001). There was a moderate, negative correlation between ‘Dangerous/unpredictable’ subscale and length of stay in Australia (*τ*_b_ = -0.25, *p* = 0.019); and a strong, negative correlation between ‘Dangerous/unpredictable’ subscale and years of education completed (*τ*_b_ = -0.33, *p* = 0.007). Moreover, there was a moderate, negative correlation between ‘I-would-not-tell-anyone’ subscale and length of stay in Australia (*τ*_b_ = -0.26, *p* = 0.017).

### Stigmatising attitudes and socio-demographic factors

The Kolmogorov–Smirnov test showed that the Personal Stigma Subscales and Social Distance Scale were not normally distributed (*p* < 0.05). Therefore, bootstrapping which is a non-parametric resampling procedure was used to test for statistical significance (Table 3). For the regression analysis with ‘I-would-not-tell-anyone’ as the dependent variable, gender (male = 1; female = 2) had a significant positive regression weight. Females were found to have higher scores for ‘I-would-not-tell-anyone’ as compared to males, after controlling for age and was retained after adjusting for multiple testing. Age and gender explained 13.9% of the variation in the ‘I-would-not-tell-anyone’ score. For the regression analysis with ‘Dangerous/unpredictable’ as the dependent variable, age had a significant negative regression weight, indicating for a unit increase in age there was a decrease in ‘Dangerous/unpredictable’ score, after controlling for gender and was retained after adjusting for multiple testing. Age and gender explained 12.5% of the variation in the ‘Dangerous/unpredictable’ score. Regression analyses with ‘Social distance’ as dependent variable did not reveal any significant differences. Age and gender explained 8.2% of the variation in the ‘Weak-not-sick’ score, and 3% of the variation in the ‘Social distance’ score.

**Table 3:** Multiple linear regression model of socio-demographic predictors of Personal Stigma Subscales and Social Distance Scale

	*B*	Bias	SE	*p*	95% CI (lower, upper)
**I-would-not-tell-anyone**					
Gender	0.77	-0.01	0.36	0.038*	0.04, 1.47
Age	-0.03	0.00	0.02	0.075	-0.05, 0.00
**Weak-not-sick**					
Gender	1.45	-0.00	0.06	0.059	-0.03, 2.94
Age	0.01	0.00	0.03	0.737	-0.05, 0.08
**Dangerous/unpredictable**					
Gender	0.25	-0.01	1.10	0.837	-1.98, 2.58
Age	-0.10	0.00	0.03	0.004*	-0.17, -0.03
**Social distance**					
Gender	-1.23	0.07	1.18	0.283	-3.53, 1.32
Age	-0.02	0.00	0.04	0.682	-0.10, 0.07

Bootstrap coefficients with 95% percentile confidence intervals. Confidence intervals and standard errors are based on 1000 bootstrap samples. *n* = 50 due to missing data. *B*: Bootstrap regression coefficient. Bias: Bootstrap bias estimate. SE: Bootstrap standard error. CI (lower, upper): confidence interval (lower limit, upper limit). * Significant using the Benjamini-Hochberg method for adjusting for multiple testing with a false discovery rate of 25%.

### Predictors of correct recognition of PTSD and selection of a cause

Odds ratios of logistic regression analyses of predictors of correct recognition of PTSD and selection of a cause as ‘likely’ are presented in Table 4. A total of 28 participants (52.8%) correctly identified Dawood/Miriam’s mental health problem as PTSD or the like. Logistic regression models did not reveal significant results for socio-demographic factors, K10 Psychological Distress Scale, Personal Stigma Subscales and Social Distance Scale.

Sixteen participants (29.6%) selected “Punishment from God” as a likely cause of developing mental illness; while 37 participants (68.5%) selected “Being a person with a weak character” as a likely cause of developing mental illness. Logistic regression analyses for the causes “Punishment from God” and “Being a person with a weak character” revealed significant results, but were not retained after adjusting for multiple testing (Table 4). Logistic regression models did not reveal significant results for K10 Psychological Distress Scale, Personal Stigma Subscales and Social Distance Scale.

**Table 4:** Logistic regression analyses of predictors of correct recognition of PTSD and selection of a cause

Variables	Correct recognition of PTSD AOR (95% CI lower, upper)	Selection of a cause as ‘likely’ AOR (95% CI lower, upper)	
		“Punishment from God”	“Being a person with a weak character”
**Socio-demographic Factors#**			
Age	0.98 (0.92, 1.05)	1.03 (0.95, 1.12)	1.07 (0.99, 1.16)
Gender	2.99 (0.68, 13.13)	6.2* (1.08, 35.36)	0.54 (0.11, 2.63)
Years of education	1.14 (0.95, 1.38)	1.10 (0.88, 1.38)	1.09 (0.90, 1.32)
Length of stay in Australia	0.99 (0.91, 1.07)	1.05 (0.96, 1.15)	0.86* (0.76, 0.97)
**Psychological Distress Scale#**			
K10	0.96 (0.90, 1.02)	0.97 (0.90, 1.05)	1.00 (0.93, 1.08)
**Personal Stigma Subscales and Social Distance Scale#**			
I-would-not-tell-anyone	0.82 (0.45, 1.50)	0.62 (0.30, 1.30)	0.74 (0.37, 1.49)
Weak-not-sick	1.00 (0.75, 1.34)	0.94 (0.66, 1.34)	0.76 (0.54, 1.07)
Dangerous/unpredictable	0.98 (0.78, 1.24)	1.01 (0.77, 1.33)	0.98 (0.75, 1.27)
Social distance	0.94 (0.77, 1.14)	1.14 (0.91, 1.42)	1.30 (0.96, 1.76)

AOR: Adjusted odds ratio. CI lower, upper: confidence interval lower limit, upper limit for odds ratio. # Adjusted for socio-demographic factors (i.e., age, gender, years of education and length of stay in Australia). * Significant at *p* < 0.05, however, none of the *p*-values retained significance using the Benjamini-Hochberg method for adjusting for multiple testing with a false discovery rate of 25%

## Discussion

The current study sought to explore the relationships between measures of mental illness stigma and various associated factors (i.e., socio-demographic factors, measure of psychological distress), as well as to determine the factors associated with MHL (i.e., recognition of mental illness, knowledge of causes) among Arabic-speaking refugee and migrant populations in Australia. The study revealed correlations between several personal stigma subscales and participants’ levels of psychological distress as measured by K10, years of education completed, age and gender.

Specifically, there was a strong positive correlation between K10 scale and ‘Dangerous/unpredictable’ subscale, indicating that an increase in the level of psychological distress was associated with increased likelihood of holding the belief that someone with mental problems is dangerous or unpredictable. A recent study among medical students in Saudi Arabia found that participants with severe psychological distress had higher scores for stigma, and emphasised that perceived stigma can be distorted by the current states of mental or emotional health [55]. Similarly, a strong negative correlation was observed between years of education completed and ‘Dangerous/unpredictable’ subscale, indicating lower levels of personal stigma among those with higher education. This is in line with prior studies that have also reported that higher education contributes to a more positive attitude towards mental illness [29, 37]. Finally, there were moderate negative correlations of the length of stay in Australia and both ‘Dangerous/unpredictable’ subscale and ‘I-would-not-tell-anyone’ subscale, indicating those who recently arrived in Australia had higher levels of such stigmatic beliefs. One possible explanation for this finding could be the high levels of psychological distress and mental illness stigma among recently arrived refugees, and prior studies have elucidated a positive association between the two [55, 56].

The current study revealed significant gender differences, where females were found to have higher levels of stigmatising attitudes towards mental illness as compared to males. A systematic review on mental illness stigma in the Arab culture also noted that Arabic-speaking males generally have more positive attitudes towards mental illness than females [29]. A prior study among Arab population in Qatar found that men had a better attitude towards mental illness than women; women were more ashamed to mention a family member with mental illness and they believed people with mental illness to be dangerous [57]. In contrast to the current finding, prior studies among a sample of the Lebanese population [58], Slovak population [59], and general Australian adult population [41] have shown lower stigmatising attitudes among females compared to males. A recent study among Arabic-speaking religious and community leaders in Australia found lower ‘Weak-not-sick’ scores for females than males that implied lower levels of stigmatising attitudes; however, the authors pointed out the existing mixed results in terms of gender differences among Arabic-speaking individuals [20].

In the current study, females had higher scores than males for ‘I-would-not-tell-anyone’ subscale, indicating that they were more likely to hold the belief that it is better not to tell others if they had a mental health problem. There are a number of plausible explanations for this study finding. Mental illness is particularly stigmatising for women in Arabic-speaking communities who are seen as the “honour” of the family [60]. Especially when others become aware that a woman is utilising mental health services, it can negatively impact marriage prospects or bring marital discord, as well as cause stigma and shame to the family [23, 32, 60]. As compared to men, a woman’s family may monitor her more closely and increase their control over her when a woman has mental illness [23]. It is also common for Arabic-speaking women to experience shame and embarrassment about seeking help regarding mental illness outside of her family [61]. Therefore, the perceived implications of a woman having a mental illness in the Arab culture may have influenced women’s attitudes toward mental illness in the current study.

A unit increase in age was associated with a decrease in ‘Dangerous/unpredictable’ score in the current study, which implied that younger people were more likely to hold the belief that someone with mental problems is dangerous or unpredictable as compared to those with older age. Age differences in the ‘Dangerous/unpredictable’ subscale were also reported for the general Australian adult population, where those aged 30–59 years had the lowest score followed by those aged ≥ 60 years and < 30 years [41]. A prior study conducted in South Sudan found that low level of education and familiarity with mental illness increased the likelihood of perceiving individuals with mental illness as dangerous, which may be some of the factors associated with younger age [37]. However, prior studies among a sample of the Slovak population [59] and Catalan population [62] have found that older age is associated with higher levels of stigmatising attitudes towards mental illness. These differences could be attributed to the use of vignette in this study, which allowed for a precise measure of personal stigma towards the character portrayed (i.e., Dawood/Miriam) rather than a measurement of general stigmatising attitudes towards mental illness as an abstract concept.

In the current study, higher odds of rating “Punishment from God” as a likely cause of developing mental illness was observed among females than males, and increase in the length of stay in Australia was associated with decreased odds of rating “Being a person with a weak character” as a likely cause of developing mental illness. However, their statistical significance was not retained after adjusting for multiple testing. The pattern of findings while not significant are consistent with previous studies. For instance, more women than men believed that mental illness is caused due to possession by evil spirits in a sample of Qatari and other Arab expatriates [57], females were found to explain mental illness by supernatural explanations more frequently than males in a study conducted in Kuwait [63], and more females than males endorsed “those who are not very religious” as a risk factor for developing PTSD in a study conducted in the United Arab Emirates [33]. Similarly, a study among resettled Iraqi refugees in Australia found that length of resettlement time positively influenced problem recognition and beliefs regarding the helpfulness of mental health treatment [22]. While consistent with previous studies, our findings failed to retain significance after adjustment for multiple testing highlighting the need for future research with larger sample size to confirm the role of gender and length of resettlement in holding stigmatising beliefs. Given stigmatising beliefs such as mental illness is God’s punishment and/or God’s will as noted in a recent systematic review [29], along with the belief that mental illness is related to personal weakness [20, 33], our findings provide additional rationale for the need for stigma reduction interventions in Arabic-speaking population groups.

### Study limitations and strengths

The limitations of the current study should be noted. These include the cross-sectional design of the study which does not allow for causal explanations, the use of self-report measures of general psychological distress, and the use of multiple hypothesis tests. The sampling technique meant that only those individuals who were most interested in participating in the MHL Program were selected. Similarly, using a vignette based on PTSD meant that only the recognition and attitudes towards PTSD were evaluated. Utilising multiple mental illness vignettes in future studies could alleviate this limitation. It should also be noted that the majority of the participants in this study were from Iraq, Syria and Lebanon, and the study findings may not be generalisable to all Arabic-speaking refugee and migrant populations in Australia. The study findings should be considered in light of limitations including the exploratory nature of the study and the small sample size (n = 53). Nonetheless, this study presents interesting findings and a starting point, particularly in light of the scarce research conducted to explore factors associated with mental illness stigma in Arabic speaking groups. Future research with a larger sample size should be undertaken to enable the confirmation of the study findings.

Notwithstanding these limitations, there were several strengths that should be mentioned. Firstly, one of the strengths of the current study was the use of a culturally adapted and valid vignette describing a fictional character with PTSD thus ensuring cultural sensitivity. Furthermore, participants were recruited from numerous non-government organisations that provided support services to Arabic-speaking migrants and/or refugees. This approach not only facilitated proportional heterogenous data collection but ensured as best an accurate representation of the Arabic-speaking community in Australia.

Our findings regarding the stigmatising attitudes and beliefs held by Arabic-speaking populations have important service implications. Most notably it is important to design stigma reduction interventions for the Arabic-speaking refugee and migrant populations in the early stage of resettlement in order to address their mental health needs and facilitate the uptake of mental health services. There is a need for more integrated and culturally informed services for these vulnerable population groups, and it is imperative to develop effective interventions to facilitate optimal adjustment for Arabic-speaking refugees and migrants, particularly those with pre-existing mental health conditions and other related factors such as poor levels of MHL and high levels of mental illness stigma [64, 65]. Research has demonstrated that community-based interventions using a group based approach such as psychotherapy programs and peer support or mentoring programs are preferred and may be beneficial to ameliorate the impact of post-migration stressors [66]. Moreover, Arabic-speaking communities often utilise avenues other than professional mental health services while seeking help for mental health difficulties, including religious and community leaders, community networks and culturally specific services [20, 39]. Therefore, it is essential to recognise the value of these informal sources in not only providing mental health care but being an entry point where stigma reduction interventions may be delivered.

## Conclusions

In summary, the study demonstrated associations between participants’ levels of psychological distress, years of education, age and gender and measures of personal stigma. While exploratory in nature with a small sample size, the study findings can be considered as a starting point to further elucidating our understanding of mental illness related stigma and a preliminary step in the development of targeted stigma reduction initiatives for Arabic-speaking refugee and migrant populations in Australia.

## Data Availability

We would like to acknowledge that data from each participant from this study cannot be shared in order to comply with the Western Sydney University Ethics policy.

## List of abbreviations

ABS: Australian Bureau of Statistics
PTSD: Post-Traumatic Stress Disorder
MHL: Mental Health Literacy
K10: Kessler Psychological Distress Scale
DSM-5: Diagnostic and Statistical Manual of Mental Disorders Fifth Edition
